# Vortioxetine in the Treatment of a Severe Depressive Episode in a Patient with ADHD Treated with Methylphenidate: A Case Report

**DOI:** 10.1192/j.eurpsy.2026.11766

**Published:** 2026-07-31

**Authors:** T. Mastelić, P. Tokić, M. Ivandić, T. Vukićević, M. Katić, M. Galetić Pavlov, T. Glavina, D. Lasić

**Affiliations:** 1 Department of Psychiatry, University Hospital of Split; 2 Department of Mental Health, Split-Dalmatia County Teaching Institute of Public Health, Split, Croatia

## Abstract

**Introduction:**

Pharmacotherapeutic options for ADHD remain limited, although it has been a growing concern for some time. Vortioxetine, a multimodal antidepressant, has shown promising results as monotherapy in the treatment of ADHD in adults. It has also demonstrated efficacy in treating anxiety and depression in adolescents. We identified two case reports describing the use of vortioxetine in treating anxiety and depression in three adolescent patients with ADHD who were also receiving methylphenidate.

**Objectives:**

This case report presents a patient with a severe depressive episode and ADHD who achieved remission of the depressive episode following treatment with vortioxetine combined with methylphenidate.

**Methods:**

Review of medical documentation.

**Results:**

The patient, a young adult in his early twenties, presented to a psychiatrist reporting loss of motivation, interest, pleasure, and energy. He experienced social withdrawal, low frustration tolerance, and hypersensitivity to noise. He reported occasional intrusive thoughts that interfered with conversation, and excessive worry about future events despite recognizing it as irrational. Academic performance was impaired, and he had difficulties falling asleep. He experienced suicidal ideation without suicidal impulses. At the moment of the examination, he clearly distanced himself from them. It was found that he has been under psychiatric treatment since middle school, treated by several psychiatrists. Previous pharmacotherapy included sertraline, mirtazapine, duloxetine, and trazodone, with limited success. He was diagnosed with ADHD three years ago. His treatment includes methylphenidate, which helps manage his ADHD symptoms, although anxiety and depressive symptoms persist. Subsequently, vortioxetine was introduced, starting at 5 mg and later increased to 10 mg, alongside methylphenidate 36 mg and alprazolam 0.25 mg. After one month of vortioxetine 5 mg, improvement was noted, including better concentration. The dose was later increased to 10 mg, and at the next visit, the patient reported a significant reduction in symptoms. After three months of treatment, the symptoms resolved completely.

**Conclusions:**

We present a case of a patient treated for ADHD and a severe depressive episode with a combination of methylphenidate and vortioxetine. The addition of vortioxetine improved concentration and led to complete remission of depressive and anxiety symptoms within three months at a dose of 10 mg. Since there is little data on this topic, we believe this case report is important. Further research is needed to clarify the role of vortioxetine as monotherapy and as an add-on therapy to methylphenidate in treating ADHD with comorbid anxiety and depression.

**Disclosure of Interest:**

None Declared

